# Supplementary material for: Causal Relationship between Aging and Anorexia Nervosa: A White-Matter-Microstructure-Mediated Mendelian Randomization Analysis
Source: Biomedicines. 2024 Aug 16;12(8):1874. doi: 10.3390/biomedicines12081874 (PMC11351342; doi:10.3390/biomedicines12081874)
Supplement: Supplementary file 1 [file biomedicines-12-01874-s001.zip › Supplementary Figure/Figure S4/Singe SNP plot of MR analysis for TL to ubm-b-1827.pdf]

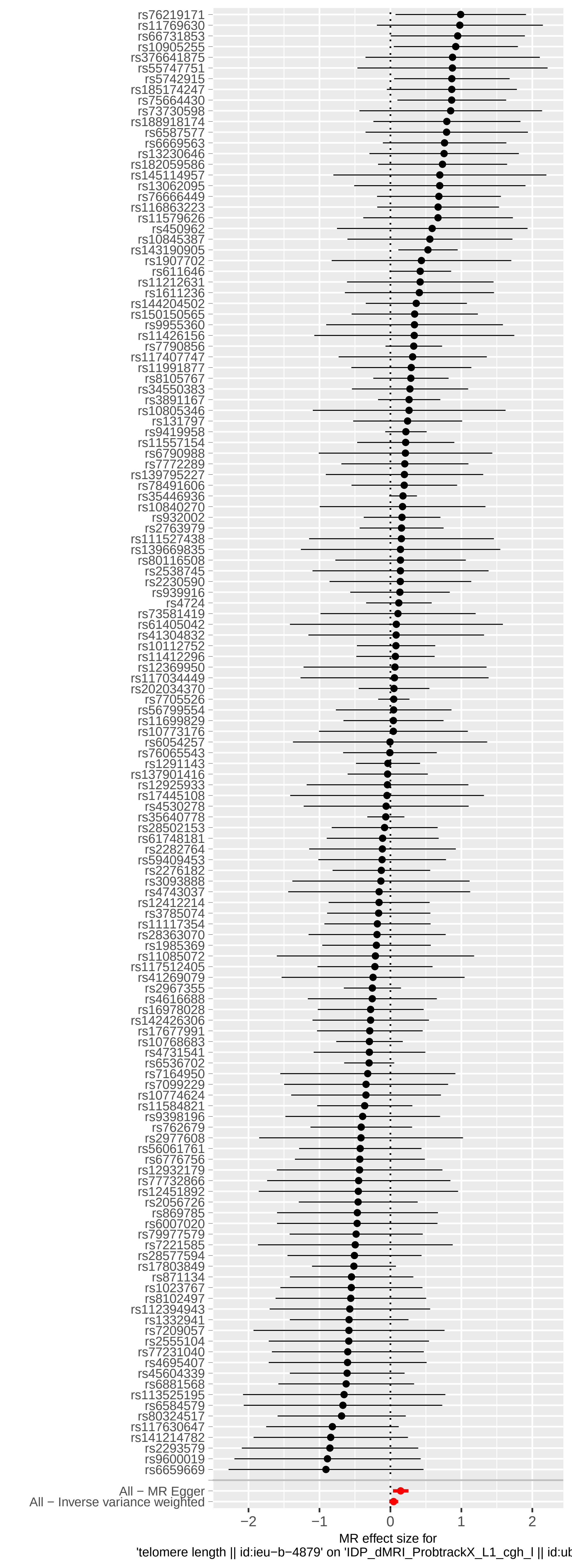
rs76219171
rs11769630
rs66731853
rs10905255
rs376641875
rs55747751
rs5742915
rs185174247
rs75664430
rs73730598
rs188918174
rs6587577
rs6669563
rs13230646
rs182059586
rs145114957
rs13062095
rs76666449
rs116863223
rs11579626
rs450962
rs10845387
rs143190905
rs1907702
rs611646
rs11212631
rs1611236
rs144204502
rs150150565
rs9955360
rs11426156
rs7790856
rs117407747
rs11991877
rs8105767
rs34550383
rs3891167
rs10805346
rs131797
rs9419958
rs11557154
rs6790988
rs7772289
rs139795227
rs78491606
rs35446936
rs10840270
rs932002
rs2763979
rs111527438
rs139669835
rs80116508
rs2538745
rs2230590
rs939916
rs4724
rs73581419
rs61405042
rs41304832
rs10112752
rs11412296
rs12369950
rs117034449
rs202034370
rs7705526
rs56799554
rs11699829
rs10773176
rs6054257
rs76065543
rs1291143
rs137901416
rs12925933
rs17445108
rs4530278
rs35640778
rs28502153
rs61748181
rs2282764
rs59409453
rs2276182
rs3093888
rs4743037
rs12412214
rs3785074
rs11117354
rs28363070
rs1985369
rs11085072
rs117512405
rs41269079
rs2967355
rs4616688
rs16978028
rs142426306
rs17677991
rs10768683
rs4731541
rs6536702
rs7164950
rs7099229
rs10774624
rs11584821
rs9398196
rs762679
rs2977608
rs56061761
rs6776756
rs12932179
rs77732866
rs12451892
rs2056726
rs869785
rs6007020
rs79977579
rs7221585
rs28577594
rs17803849
rs871134
rs1023767
rs8102497
rs112394943
rs1332941
rs7209057
rs2555104
rs77231040
rs4695407
rs45604339
rs6881568
rs113525195
rs6584579
rs80324517
rs117630647
rs141214782
rs2293579
rs9600019
rs6659669
All − MR Egger
All − Inverse variance weighted
−2
−1
0
1
2
MR effect size for
'telomere length || id:ieu−b−4879' on 'IDP_dMRI_ProbtrackX_L1_cgh_l || id:ub
